# Proper training and use of ultrasonography facilitates lumbar puncture

**DOI:** 10.1186/s13049-017-0466-x

**Published:** 2017-12-20

**Authors:** Geert-Jan van Geffen, Rein Ketelaars, Jörgen Bruhn

**Affiliations:** 1 Department of Anesthesiology, Pain and Palliative Medicine, Radboud university medical center, Nijmegen, The Netherlands; 2 Helicopter Emergency Medical Service, Lifeliner 3, Radboud university medical center, Nijmegen, The Netherlands

**Keywords:** Ultrasonography, Spinal puncture, Emergency service, Hospital, Emergency medicine

## Abstract

With great interest, we read the study of Line Dussourd et al. concluding that ultrasonography allows better identification of anatomical structures before performing a lumbar puncture. We cannot concur with the conclusions of the study because the authors did not visualize the conus medullaris directly, nor did they assess the individual intervertebral levels. In our commentary, we make some suggestions for improvement using ultrasound to locate the optimal site for a lumbar puncture. We do agree that neuraxial ultrasound is of great benefit for the performance of lumbar punctures. Proper training and applying the correct technique, however, is necessary for obtaining all benefits ultrasonography offers.

To the editor:

Sir,

With interest, we read the article of Dussourd et al. [1]. Although we agree with their conclusion that ultrasonography allows better identification of anatomical structures before lumbar puncture, we cannot concur with their statement that ultrasound identified the best lumbar puncture site under the conus medullaris. The authors did not visualize the conus medullaris directly, nor did they assess the individual intervertebral levels.

To improve the accuracy of intervertebral space identification in preparation for a lumbar puncture a pre-procedure spinal ultrasound scan should be performed identifying the individual intervertebral levels. This may be performed by counting spinous processes or laminae upward from the sacrum. Using this method, ultrasound may accurately identify the correct intervertebral space in 76% of the cases [2].

Moreover, identifying a lumbar interlaminar space is not easy and it is hard to achieve competency in all aspects of spinal ultrasonography [3]. Trainees in anesthesiology were only considered competent after performing 60 supervised scans. Therefore, we doubt whether the participating emergency physicians obtained all necessary skills to successfully identify the correct lumbar intervertebral space after a training which did not exceed 30 min.

Neuraxial ultrasound improves the efficacy of neuraxial techniques. A recent meta-analysis showed the combined risk ratio of technical failure in lumbar neuraxial procedures was 0.51 (95% CI, 0.32–0.80) when ultrasound guidance is used compared to palpation. In addition, ultrasound guidance results in a lower number of needle passes required for success [4].

## Conclusions

In conclusion, we do agree that neuraxial ultrasound is of great benefit for the performance of lumbar punctures. Proper training and applying the correct technique, however, is necessary for obtaining all benefits ultrasonography offers.

Geert-Jan van Geffen,

Rein Ketelaars,

Jörgen Bruhn.
